# Performance of Affinity-Improved DARPin Targeting HIV Capsid Domain in Interference of Viral Progeny Production

**DOI:** 10.3390/biom11101437

**Published:** 2021-09-30

**Authors:** Kanokporn Sornsuwan, Weeraya Thongkhum, Thanathat Pamonsupornwichit, Tanawan Samleerat Carraway, Suthinee Soponpong, Supachai Sakkhachornphop, Chatchai Tayapiwatana, Umpa Yasamut

**Affiliations:** 1 Division of Clinical Immunology, Department of Medical Technology, Faculty of Associated Medical Sciences, Chiang Mai University, Chiang Mai 50200, Thailand; kanokporn_sornsuwan@cmu.ac.th; 2 Center of Biomolecular Therapy and Diagnostic, Faculty of Associated Medical Sciences, Chiang Mai University, Chiang Mai 50200, Thailand; weeraya.t@cmu.ac.th (W.T.); thanathat.cmu@gmail.com (T.P.); pear121000@gmail.com (S.S.); 3 Center of Innovative Immunodiagnostic Development, Faculty of Associated Medical Sciences, Chiang Mai University, Chiang Mai 50200, Thailand; 4 Division of Clinical Microbiology, Department of Medical Technology, Faculty of Associated Medical Sciences, Chiang Mai University, Chiang Mai 50200, Thailand; tsamleerat@gmail.com; 5 Research Institute for Health Sciences, Chiang Mai University, Chiang Mai 50200, Thailand; ssakkhachornphop@yahoo.com

**Keywords:** ankyrin, capsid, HIV-1 assembly, anti-HIV-1 molecule, HIV-1 drug resistance

## Abstract

Previously, a designed ankyrin repeat protein, Ank^GAG^1D4, was generated for intracellular targeting of the HIV-1 capsid domain. The efficiency was satisfactory in interfering with the HIV assembly process. Consequently, improved Ank^GAG^1D4 binding affinity was introduced by substituting tyrosine (Y) for serine (S) at position 45. However, the intracellular anti-HIV-1 activity of Ank^GAG^1D4-S45Y has not yet been validated. In this study, the performance of Ank^GAG^1D4 and Ank^GAG^1D4-S45Y in inhibiting wild-type HIV-1 and HIV-1 maturation inhibitor-resistant replication in SupT1 cells was evaluated. HIV-1 p24 and viral load assays were used to verify the biological activity of Ank^GAG^1D4 and Ank^GAG^1D4-S45Y as assembly inhibitors. In addition, retardation of syncytium formation in infected SupT1 cells was observed. Of note, the defense mechanism of both ankyrins did not induce the mutation of target amino acids in the capsid domain. The present data show that the potency of Ank^GAG^1D4-S45Y was superior to Ank^GAG^1D4 in interrupting either HIV-1 wild-type or the HIV maturation inhibitor-resistant strain.

## 1. Introduction

Human immunodeficiency virus (HIV) infection remains a major health problem worldwide. Highly active antiretroviral therapy (HAART) is currently used to sustain HIV suppression and recover the immune function of patients [1,2]. Despite success in terms of improved clinical symptoms, adverse drug effects from using HAART have been reported. Therefore, alternative strategies have been developed for HIV therapy [3]. Several intrabodies have been designed to target the viral HIV-1 protein, e.g., scFvD8 [4], GPI scFv-X5 [5], and scFv 183-H12-5C [6], which were generated to inhibit HIV-1 replication in infected cells. However, cytoplasmic reducing conditions halted their development, since proper folding and stability requires disulfide bond formation.

Accordingly, the attempt to construct a disulfide bond-independent scaffold might be promising for HIV-1 therapy. An alpha repeat (αRep) protein has been designed to target HIV-1 Gag. This αRep exhibits activity by impairing the viral packaging and maturation process [7,8]. Another type of disulfide bond-free scaffold is called designed ankyrin repeat protein (DARPin), which is based on natural ankyrin [9]. This building block provides the properties of ankyrin in protein–protein interactions involved in several cellular activities [10,11,12]. The advantages of DARPin include high stability and solubility. Furthermore, resistance in the protease and reducing cytoplasmic environment may make ankyrin an intracellular therapeutic molecule [10]. According to these advantages, DARPins were designed to overcome several limitations when using immunoglobulins as therapeutics agents [13,14,15,16]. In addition, the DARPins have been reported to have a role in HIV inhibition. CD4-specific DARPins [17] and HIV gp120-specific DARPins [18] were designed to block HIV-1 entry. However, their efficiency was reduced by unwanted side effects [19] and mutation in the HIV envelope [20].

Besides the extracellular anti-HIV-1 DARPins, we reported an intracellular anti-HIV-1 DARPin, Ank^GAG^1D4, which specifically targets the N-terminus of the HIV-1 capsid protein [21]. Ank^GAG^1D4 provides anti-HIV-1 activity through interference with HIV Gag multimerization, an important step in HIV assembly. This ankyrin reduces the permissiveness of HIV-1 production in HIV-1-infected SupT1 cells [22]. In addition, Ank^GAG^1D4 has broad-spectrum antiviral activity against an HIV-1 circulatory strain that carries a mutation in the N-terminus capsid [23]. However, the anti-HIV-1 activity of Ank^GAG^1D4 was mediocre, especially in the late stage of infection [24]. Computational analysis and calculation of van der Waals (vdW) forces indicate the choices of key amino acid residues in ankyrin sequence [25]. An evaluation of the binding activity and affinity using an enzyme linked immunosorbent assay (ELISA)-modified method and bio-layer interferometry (BLI) showed that substitution of serine (S) at position 45 with tyrosine (Y), forming Ank^GAG^1D4-S45Y, leads to increased affinity against the HIV-1 capsid domain. Enhanced binding affinity of Ank^GAG^1D4 might provide complete HIV-1 inhibition.

The emergence of drug-resistant strains is another important obstacle in HIV-1 therapy. Mutations in the genes involved with antiretroviral drug target sites are continuously reported [26,27], resulting in the failure of HAART. Nowadays, several HIV-1 drugs and inhibitors have been developed in order to overcome this problem [28]. Capsid-targeting inhibitors represent one interesting compound, which work by interfering in the late stage of the HIV-1 life cycle, assembly and maturation [29,30]. The HIV-1 maturation inhibitor (MI) is a class of anti-HIV-1 compound that blocks proteolytic cleavage of the Gag protein, resulting in non-infectious virions. MI can be divided into two classes; betulinic acid-based and pyridone-based MI. The betulinic acid-based MI, bevirimat (BVM), blocks HIV-1 maturation by interrupting CA-SP1 cleavage [31]. According to the resistance-conferring mutation on the Gag protein, a BVM derivative, C28-BVM, was further developed [32]. The second class of MI, PF46396, exhibits antiretroviral activity in HIV-1 laboratory strain and HIV-1 circulatory isolates. However, HIV-1 resistance against both classes of MI has been reported [33,34,35]. These data indicate that even though new anti-HIV-1 agents were developed, it is not enough to inhibit HIV-1 replication. As the target region of Ank^GAG^1D4 is distinctive from that of MI, Ank^GAG^1D4 is expected to inhibit the assembly process of the HIV-1 MI-resistant strain.

This study was aimed at investigating the anti-HIV activity of binding affinity-enhanced Ank^GAG^1D4 in infected SupT1 cells. In addition, the role of the Ank^GAG^1D4 in HIV-1 maturation inhibitor resistant (MIR) strain was addressed. The HIV-1 NL4-3 MIR_CAI201V_ virus, carrying a mutation on the CA-SP1 junction on Gag protein, was used as a model. Regarding our results, the binding affinity-improved Ank^GAG^1D4 had increased antiviral activity against wild-type (WT) and MIR viruses.

## 2. Materials and Methods

### 2.1. Cell Lines and Plasmid

SupT1 cells (ATCC) were cultured in Roswell Park Memorial Institute (RPMI) 1640 medium (Gibco) supplemented with 10% heat-inactivated fetal bovine serum (FBS), 100 U/mL of penicillin (Gibco), 100 µg/mL of streptomycin (Gibco), and 2 mM of L-glutamine (Gibco). HEK293T cells were cultured in Dulbecco’s Modified Eagle Medium (DMEM) supplemented with 10% heat-inactivated FBS (Gibco), 100 U/mL of penicillin (Gibco), 100 µg/mL of streptomycin (Gibco), and 2 mM of L-glutamine (Gibco).

pNL4-3 plasmid, the infectious HIV-1 NL4-3 molecular clone (NIH), was used to produce the HIV-1 NL4-3 laboratory strain virus. Additionally, mutagenesis was performed on this plasmid to generate a clone of the HIV-1 MIR virus.

### 2.2. Preparation of HIV-1 Virions

HIV-1 NL4-3 viral stock was produced as previously described [36]. Briefly, 5 × 10^6^ HEK293T cells were seeded in a 10 cm^2^ dish containing 10% heat-inactivated FBS-DMEM. At 70% cell confluence, cells were transfected with 5 µg of pNL4-3 plasmid using Lipofectamine^TM^ LTX reagent and PLUS^TM^ reagent (Thermo Fisher Scientific, Waltham, MA, USA). After 48 h post-transfection, culture supernatant containing virus was harvested, centrifuged at 335× *g* for 5 min and filtrated through a 0.45 µm filter membrane to remove unwanted particles. The viral stock was aliquoted and kept at −80 °C. HIV-1 viral titer was determined by HIV viral load assay using COBAS AmpliPrep/COBAS Taqman HIV-1 test (Roche, Basel, Switzerland).

### 2.3. Construction of pNL4-3 MIR_CAI201V_ Plasmid and Preparation of HIV-1 Maturation Inhibitor Resistant (MIR) Virus

In order to compare the function of Ank^GAG^1D4 and Ank^GAG^1D4-S45Y in HIV-1 MIR virus production, HIV-1 NL4-3 MIR_CAI201V_ was generated. Mutation at position 201 on CA-CTD from isoleucine (I) to valine (V), CAI201V, confers resistance of HIV-1, in both clade B and C, against PF-46396, and partial resistance to BVM [33,35]. To construct the molecular clone of HIV-1, NL4-3 MIR_CAI201V_, mutagenesis was performed on pNL4-3 plasmids using a QuickChange Lightening Site-Directed Mutagenesis Kit (Agilent Technologies, Santa Clara, CA, USA). The synthesized oligonucleotides used in this experiment were as follows: Fwd_CAI201V: 5′cgaacccagattgtaagactgtgttaaaagcattgggacca-3′; Rev_CAI201V: 5′tggtcccaatgcttttaacacagtcttacaatctgggttcg-3′. The mutated plasmid was transformed into XL-1 blue competent *E. coli* cells for plasmid amplification. The plasmid-harboring XL-1 blue cells were grown on Luria–Bertani (LB) agar supplemented with 100 µg/mL of ampicillin, at 37 °C for 16 h. A bacterial colony was picked and further cultured in super broth (SB) supplemented with 100 µg/mL of ampicillin at 37 °C for 16 h. After culturing, the plasmid was extracted and purified using a Geneaid™ Midi Plasmid Kit (Geneaid Biotech, New Taipei, Taiwan). To confirm the corrected mutagenesis, pNL4-3 MIR_CAI201V_ was subjected to plasmid sequencing analysis.

pNL4-3 MIR_CAI201V_ was used for HIV-1 NL4-3 MIR_CAI201V_ viral production. This plasmid was transfected into HEK293T cells using MirusTransITX2 (Mirus Bio, Madison, WI, USA). After 48 h post-transfection, culture supernatant containing virus was harvested, centrifuged at 335× *g* for 5 min, and filtrated through 0.45 µm filter membrane to remove unwanted particles. The viral stock was aliquoted and kept at −80 °C. HIV-1 viral titer was determined by HIV viral load assay using COBAS Ampliprep/COBAS Taqman HIV-1 test (Roche, Basel, Switzerland).

### 2.4. Generation of SupT1 Cells Stably Expressing Ankyrin Protein, Ank^GAG^1D4-EGFP, Ank^GAG^1D4-S45Y-EGFP, and Ank^A3^2D3-EGFP by Lentiviral Gene Transferring Method

To generate SupT1 cells stably expressing ankyrin protein, 1 × 10^5^ of SupT1 cells were transduced with VSV-G pseudotyped lentiviral vector at a multiplicity of infection (MOI) of 1, with the addition of 5 ug/mL polybrene. Each VSV-G pseudotyped lentiviral vector included VSVG–CGW–Myr (+) Ank^GAG^1D4-EGFP, VSVG–CGW–Myr (+) Ank^GAG^1D4-S45Y-EGFP, and VSVG–CGW–Myr (+) Ank^A3^2D3-EGFP. These cells were spinoculated at 2500× *g* for 1.30 h, and further cultured for 16 h. After incubation, these cells were washed 3 times with RPMI 1640 medium and cultured in 10% heat-inactivated FBS-RPMI 1640. To evaluate ankyrin expression in SupT1 cells, EGFP-positive cells were observed under an inverted fluorescence microscope (Zeiss Axio Observer-Colibri 7) and the percentage of EGFP-positive cells was determined by a CyAn^TM^ ADP flow cytometer (Beckman Coulter, Brea, CA, USA). SupT1 stable cells were sorted by a BD FACSMelody^TM^ cell sorter (BD biosciences, Franklin Lakes, NJ, USA) to obtain the comparable expression level of ankyrin. 2.6. Evaluation of CD4 surface expression on SupT1 was done on cells stably expressing ankyrin protein. To test whether overexpression of ankyrin in SupT1 cells interferes with CD4 expression on the cell surface, CD4 protein was examined by immunofluorescence staining. SupT1 cells and SupT1 stable cells were washed twice with phosphate-buffered saline (PBS) and incubated in 10% human AB serum-PBS on ice for 30 min. After incubation, the cells were stained with APC-conjugated mouse anti-human CD4 antibody (Immunotools, Friesoythe, Germany) and placed on ice for 30 min. Next, cells were washed 3 times with FACS buffer solution and resuspended in fixation buffer (1% paraformaldehyde in PBS). CD4-positive cells were analyzed by a BD Accuri^TM^C6 cytometer (BD biosciences, Franklin Lakes, NJ, USA).

### 2.5. Determination of Subcellular Localization of Ankyrin Proteins in SupT1 Cells

To determine subcellular localization of ankyrin proteins, ankyrin-EGFP-expressing SupT1 cells were observed under confocal fluorescence microscopy. SupT1 cells and ankyrin-EGFP-expressing SupT1 cells were centrifuged at 335× *g* for 5 min, then resuspended in RPMI 1640 medium. A total of 1 × 10^6^ cells of SupT1 cells or ankyrin-EGFP-expressing SupT1 cells were seeded to poly-L lysine-precoated cover slips. Cells were incubated in humidified a 5% CO_2_ atmosphere incubator at 37 °C for 10 min. Cells were subsequently incubated in fixation buffer (4% paraformaldehyde in PBS) at room temperature for 15 min. After twice washing with PBS, cells were stained with a 1:1000 dilution of CellMask^TM^ Deep red membrane staining (Thermo Fisher Scientific, Waltham, MA, USA) and 1:1000 dilution of Hoechst 33342 (Thermo Fisher Scientific, Waltham, MA, USA) in RPMI 1640 medium at 37 °C for 10 min. Cell imaging was performed using Nikon C2 plus confocal fluorescence microscopy (Nikon, TYO, Japan) at 63× magnification. Excitation wavelengths were 405 nm for Hoechst 33342, 488 nm for EGFP, and 560 nm for CellMask^TM^ Deep red membrane staining.

### 2.6. HIV-1 Challenge

SupT1 cells and ankyrin-expressing SupT1 cells were incubated with 10 MOI of HIV-1 NL4-3 or NL4-3 MIR_CAI201V_ virus. In this experiment, SupT1 cells without exogenous ankyrin expression and SupT1 cells expressing irrelevant ankyrin (Myr (+) Ank^A3^2D3-EGFP) were used as controls. After incubation, these cells were washed 3 times with RPMI 1640 medium, then cultured in 10% heat-inactivated FBS-RPMI 1640 medium. During the culture, cells were observed for syncytium formation under inverted microscopy. Cells were subcultured every 2 days, and culture supernatants were collected at days 3, 5, 7, 9, 11, 13, 17, and 21 post-infection. Collected culture supernatants were centrifuged to removed debris and unwanted particles. Culture supernatant was kept at −80 °C for HIV-1 p24 and viral load assay.

### 2.7. Evaluation of HIV-1 p24 and Viral Load

The level of HIV-1 capsid (p24) in culture supernatant was evaluated using a Genscreen^TM^ Ultra p24 ELISA kit (Bio-Rad, Marnes-la-Conquette, PAR, France). The viral particles in culture supernatant were lysed by 1% Triton-X 100 prior to the assay. Culture supernatants were added to a well precoated with monoclonal antibody against HIV-1 p24. After incubation and washing, biotinylated anti-HIV-1 p24 polyclonal antibody was added. The reaction was incubated at room temperature for 30 min, then washed. Next, HRP-conjugated streptavidin was added to the well, and the reaction was incubated for 30 min at room temperature. After incubation and washing, the reaction was detected by adding chromogenic substrate, and stopped at 30 min with 1 N sulfuric acid solution. The absorbances were read using a microplate reader at 450 nm, and calculated for HIV-1 p24 levels using HIV-1 p24 standard curve. To further determine the viral production, culture supernatants at 13 days post- infection were subjected to HIV viral load assay. The level of HIV virion in culture supernatant was evaluated using reverse transcription quantification polymerase chain reaction (RT-qPCR) by COBAS Ampliprep/COBAS Taqman HIV-1 test (Roche, Basel, Switzerland).

### 2.8. Analysis of HIV-1 Capsid Sequence

The sequence analysis of the HIV-1 capsid was modified from a previously described method [22]. In brief, viral RNA was extracted from culture supernatant using a QIAamp Viral RNA Mini Kit (Qiagen, Hilden, Germany). To generate cDNA encoding the HIV-1 capsid, extracted RNA was used as a template for reverse transcription PCR (RT-PCR) using a Superscript III One-step RT-PCR system (Invitrogen, Friesoythe, Germany). The PCR reaction contained a pair of oligonucleotides, (FWD_RIHES_p24: 5′-ggatagaggtaaaagacaccaaggaagc-3′; REV_RIHES_p24: 5′-ctcattgcctcagccaaaacccttgc-3′), and PCR product was purified using a GENEJET PCR purification kit (Thermo Fisher Scientific, Waltham, MA, USA). The purified PCR product was subjected to DNA sequencing with the same oligonucleotides with RT-PCR. To analyze sequencing results, HIV-1 capsid sequence was aligned against HIV-1 NL4-3 WT using SnapGene software version 2.8.3 (GSL Biotech, San Diego, CA, USA).

### 2.9. Statistical Analysis

The data are presented as the mean ± S.D. from 3 replicate experiments. Statistical analysis was performed using unpaired *t*-test. Differences were considered significant at *p* < 0.05 (indicated with asterisks).

## 3. Results

### 3.1. Expression of Ankyrin Protein Did Not Interfere with Cell-Surface CD4

**Figure 1:**
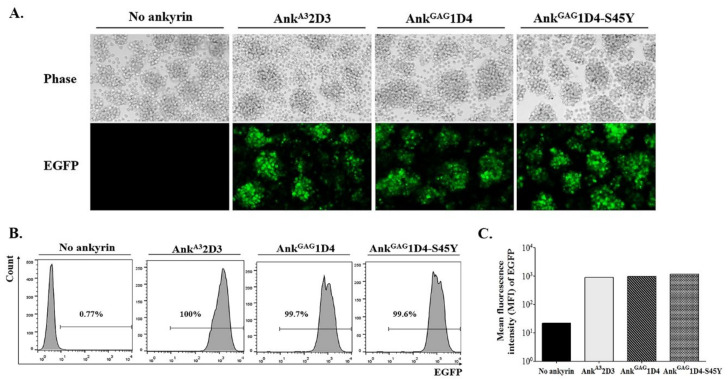
Establishment of SupT1 cells stably expressing ankyrin proteins. SupT1 cells were transduced with 1 MOI of VSV-G pseudotyped virus carrying gene encoding the ankyrin protein with EGFP fusion. (**A**) After 48 h post-transduction, EGFP-positive cells were observed under fluorescent microscopy. Cell imaging was done at 20× magnification with the same exposure time using Axio Observer 7. (**B**) After cell sorting, the level of ankyrin expression in SupT1 cells was investigated by flow cytometry. EGFP signal indicates ankyrin-expressing SupT1 cells. (**C**) EGFP intensity in ankyrin-expressing SupT1 cells shown in a bar graph. No ankyrin, Ank^A3^2D3, Ank^GAG^1D4, and Ank^GAG^1D4-S45Y represent SupT1 cell control, SupT1 cells expressing Myr (+) Ank^A3^2D3-EGFP, Myr (+) Ank^GAG^1D4-EGFP, and Myr (+) Ank^GAG^1D4-S45Y-EGFP, respectively.

**Figure 2:**
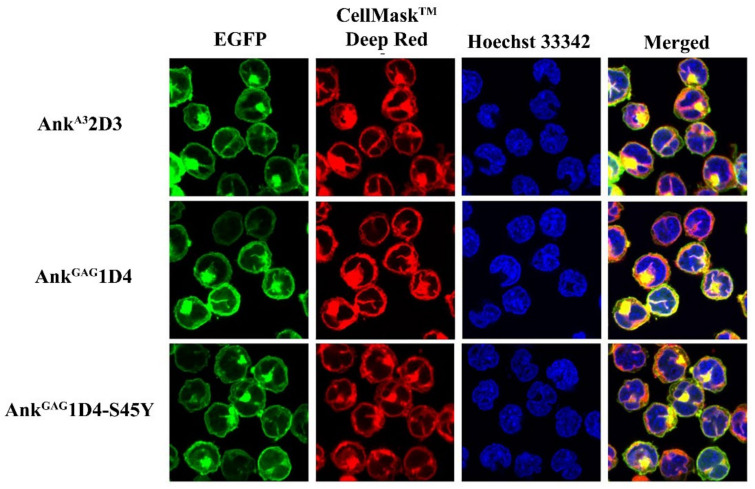
Subcellular localization of ankyrins in SupT1 cells. Ankyrin-EGFP expressing SupT1 cells were stained with plasma membrane dye, CellMask^TM^ Deep Red. Nuclei were stained with Hoechst 33342. Confocal imaging was done at 60× magnification using Nikon C2 plus confocal fluorescence microscopy. Green represents EGFP-tagging ankyrins. Blue indicates nucleus, and red shows the plasma membrane of SupT1 cells. AnkA32D3, Ank^GAG^1D4, and Ank^GAG^1D4-S45Y refer to SupT1 cells expressing Ank^A3^2D3, Ank^GAG^1D4, and Ank^GAG^1D4-S45Y, respectively.

**Figure 3:**
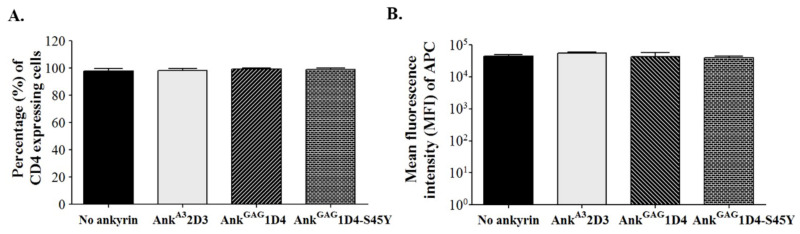
Surface CD4 expression on ankyrin-expressing SupT1 cells. Ankyrin-expressing SupT1 cells were stained with APC conjugated anti-human CD4 antibody and analyzed by flow cytometry. (**A**) Percentage of CD4 positive cells represented in a bar graph. (**B**) Mean fluorescence intensity of APC indicates the level of CD4 expression on SupT1 cells. Data represent mean ± SD from three independent experiments. No ankyrin, Ank^A3^2D3, Ank^GAG^1D4, and Ank^GAG^1D4-S45Y represent SupT1 cell control, SupT1 cells expressing Myr (+) Ank^A3^2D3-EGFP, Myr (+) Ank^GAG^1D4-EGFP, and Myr (+) Ank^GAG^1D4-S45Y-EGFP, respectively.

To generate SupT1 stable cells, SupT1 cells were transduced with VSV-G pseudotyped lentivirus. Each lentivirus vector carries the gene encoding N-terminus myristoylated ankyrin protein with enhanced green fluorescence protein (EGFP) fusion, including Myr (+) Ank^A3^2D3-EGFP, Myr (+) Ank^GAG^1D4-EGFP, and Myr (+) Ank^GAG^1D4-S45Y-EGFP. After 48 h post-transduction, SupT1 cells were observed to be EGFP positive under fluorescence microscopy (Figure 1A). Since a comparable expression level of ankyrin is required to verify their anti-HIV-1 activity in infected cells, these transduced SupT1 cells were sorted. After cell sorting, the percentage of EGFP-positive cells in SupT1 stable cells was approximately 100%, with a comparable level of ankyrin (Figure 1B,C). In addition, subcellular localization of ankyrin proteins in SupT1 cells was determined under confocal microscopy. The result showed that EGFP tagging ankyrins were colocalized with a plasma membrane dye, suggesting their targeting to inner leaflet of cell membrane as a result from the N-terminus myristoylation signal (Figure 2). To examine whether the expression of ankyrin interfered with the HIV receptor, CD4 expression on the surface of SupT1 stable cells was measured by flow cytometry. The results demonstrated that the number of CD4-positive cells in ankyrin-expressing and control SupT1 cells was similar (Figure 3A). The mean fluorescence intensity of CD4 in SupT1 cells and ankyrin-expressing SupT1 cells (Myr (+) Ank^A3^2D3-EGFP, Myr (+) Ank^GAG^1D4-EGFP, and Myr (+) Ank ^GAG^1D4-S45Y-EGFP) was 4.47 × 10^4^, 5.58 × 10^4^, 4.31 × 10^4^, and 3.96 × 10^4^, respectively (Figure 3B). These data suggest that the expression of N-terminus myristoylated ankyrin protein did not alter the level of CD4 on the cell surface of SupT1 cells.

### 3.2. Ank^GAG^1D4-S45Y Provides More Protection against HIV-1-Mediated Cell Death

**Figure 4:**
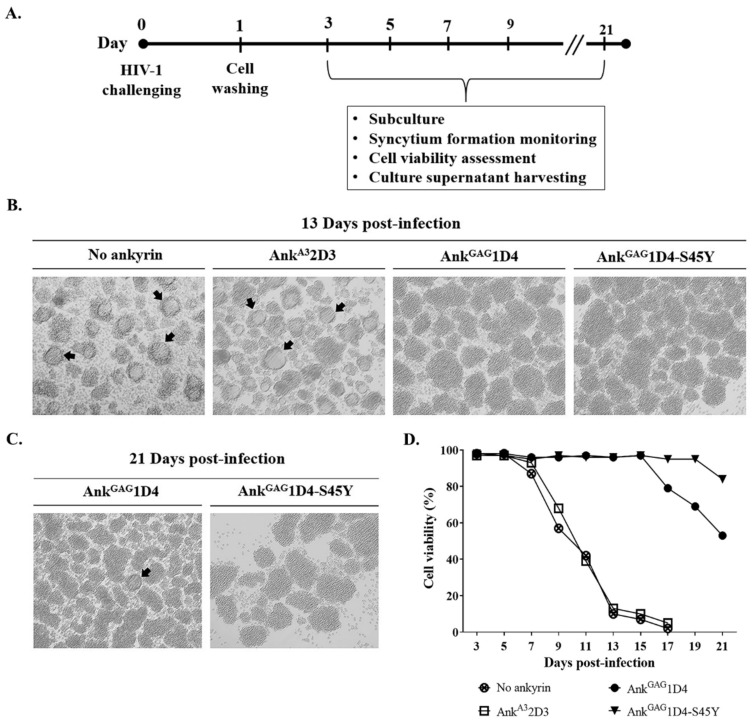
Syncytium cell formation and cell viability of HIV-1-infected SupT1 cells expressing ankyrins. (**A**) SupT1 cells and SupT1 stable cells were infected with HIV-1 NL4-3 laboratory strain at 10 MOI. Infected cells were cultured and processed as shown. After HIV-1 challenge, cells were subcultured every 2 days. (**B**) At 13 days post-infection, syncytium formation in infected cells was observed under microscopy. Cell imaging was done at 20× magnification using Axio Vert.A1. Arrows point to syncytium cells. (**C**) Syncytium formation in infected SupT1/Myr (+) Ank^GAG^1D4-EGFP and SupT1/Myr (+) Ank^GAG^1D4-S45Y-EGFP was continuously observed until 21 days post-infection. (**D**) Cell viability of infected cells was determined using the Trypan blue exclusion method. No ankyrin, Ank^A3^2D3, Ank^GAG^1D4, and Ank^GAG^1D4-S45Y represent SupT1 cell control, SupT1 cells expressing Myr (+) Ank^A3^2D3-EGFP, Myr (+) Ank^GAG^1D4-EGFP, and Myr (+) Ank^GAG^1D4-S45Y-EGFP, respectively.

To investigate the antiviral activity of ankyrin targeting the HIV-1 capsid, SupT1 and SupT1 stable cells (SupT1/Myr (+) Ank^A3^2D3-EGFP, SupT1/Myr (+) Ank^GAG^1D4-EGFP, and SupT1/Myr (+) Ank^GAG^1D4-S45Y-EGFP) were infected with HIV-1 NL4-3 laboratory strain virus at an MOI of 10. A schematic representation of the experiments is shown in Figure 4A. Following HIV-1 infection, the cytopathic effect in infected cells was observed under inverted microscopy (Figure 4B,C). Syncytium formation in SupT1 and SupT1/Myr (+) Ank^A3^2D3 was detected early at 5 days post-infection (Figure S1). The parental Myr (+) Ank^GAG^1D4 delayed the formation of syncytium cells in infected cells to 21 days post-infection, while no syncytium cells were observed in SupT1 cells expressing Myr (+) Ank^GAG^1D4-S45Y-EGFP. In addition, cell viability was monitored by the trypan blue exclusion method. The cell viability of HIV-1-infected SupT1 and SupT1/Myr (+) Ank^A3^2D3-EGFP was dramatically decreased at 9 days post-infection, and they were entirely dead at day 17 (Figure 4D). On the other hand, SupT1/Myr (+) Ank^GAG^1D4-EGFP and SupT1/Myr (+) Ank^GAG^1D4-S45Y-EGFP had extended cell viability to 21 days with a greater percentage of viable cells in the latter.

### 3.3. Ank^GAG^1D4-S45Y Improves Antiviral Activity Than Parental Ankyrin in HIV-1-Infected SupT1 Cells

**Figure 5:**
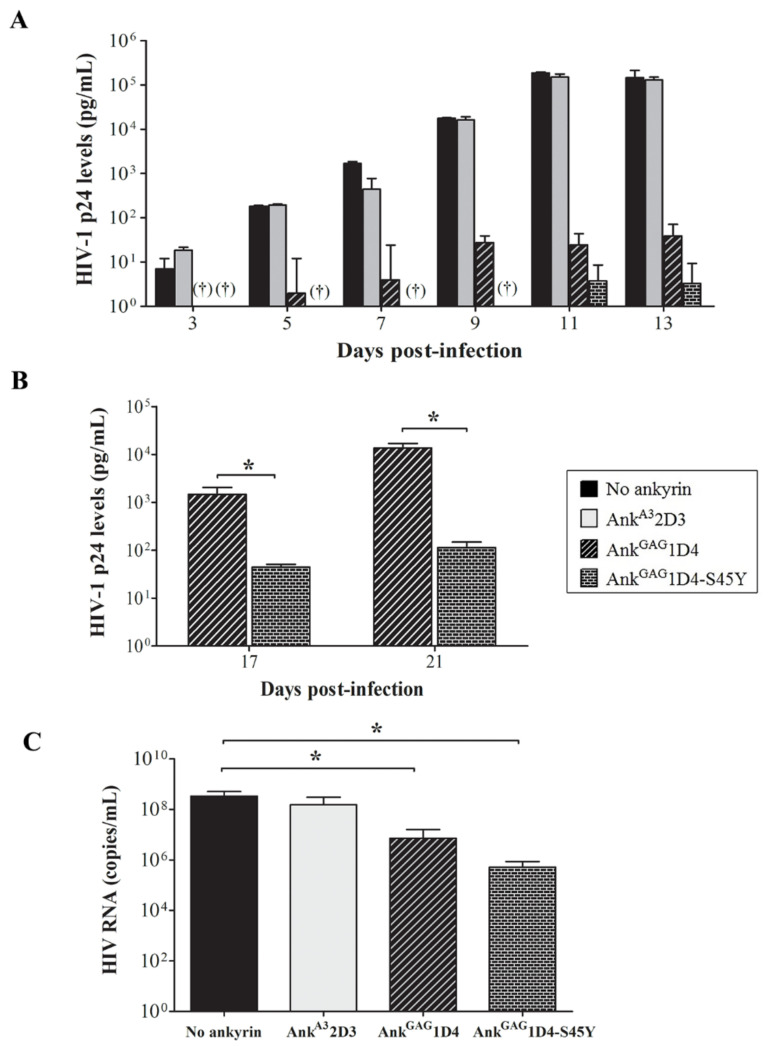
HIV replication in 10 MOI of HIV-1-infected SupT1 cells expressing binding affinity-enhanced Ank^GAG^1D4. After HIV-1 challenge, cells were subcultured every 2 days. Cultured supernatant was collected at 3, 5, 7, 9, 11, 13, 17, and 21 days post-infection, then assayed to evaluate HIV-1 production. (**A**,**B**) HIV-1 p24 levels were determined using p24 ELISA. † Indicates undetectable HIV-1 p24 level. (**C**) HIV RNA copies were determined at 13 days post-infection using HIV viral load assay. Bar graph shows HIV RNA copies from viral load assay. Data represent mean ± SD from triplicate wells. * *p* ≤ 0.05 using unpaired t-test. No ankyrin, Ank^A3^2D3, Ank^GAG^1D4, and Ank^GAG^1D4-S45Y represent SupT1 cell control, SupT1 cells expressing Myr (+) Ank^A3^2D3-EGFP, Myr (+) Ank^GAG^1D4-EGFP, and Myr (+) Ank^GAG^1D4-S45Y-EGFP, respectively.

HIV-1 production in SupT1 cells and ankyrin-expressing SupT1 cells was evaluated using HIV-1 p24 ELISA. The level of p24 in SupT1 cells control was detected at 3 days post-infection, and continuously increased to 1.46 × 10^5^ pg/mL at 13 days (Figure 5A). SupT1/Myr (+) Ank^A3^2D3, an irrelevant ankyrin, showed continuously increased p24 levels to 1.30 × 10^5^ pg/mL at 13 days and was not different from infected the SupT1 cell control. Myr (+) Ank^GAG^1D4-EGFP and Myr (+) Ank^GAG^1D4-S45Y-EGFP showed higher anti-HIV-1 potency based on 1000-fold lower HIV-1 production than the SupT1 cell control. However, at day 17, superior anti-HIV-1 activity of Myr (+) Ank^GAG^1D4-S45Y-EGFP was indicated (Figure 5B). The level of HIV-1 p24 in Myr (+) Ank^GAG^1D4-EGFP and Myr (+) Ank^GAG^1D4-S45Y-EGFP was 1.47 × 10^3^ and 44.36 pg/mL, respectively. HIV viral load assay confirmed the ability of Myr (+) Ank^GAG^1D4-EGFP and Ank^GAG^1D4-S45Y-EGFP to inhibit viral replication, as the HIV RNA copy number was lower than in the SupT1 cell control and irrelevant ankyrin (Figure 5C). Moreover, HIV viral load confirmed that the number of RNA copies in SupT1/Myr (+) Ank^GAG^1D4 -S45Y-EGFP was 5.12 × 10^5^ copies/mL, and in SupT1/Myr (+) Ank^GAG^1D4-EGFP was 7.20 × 10^6^ copies/mL.

**Figure 6:**
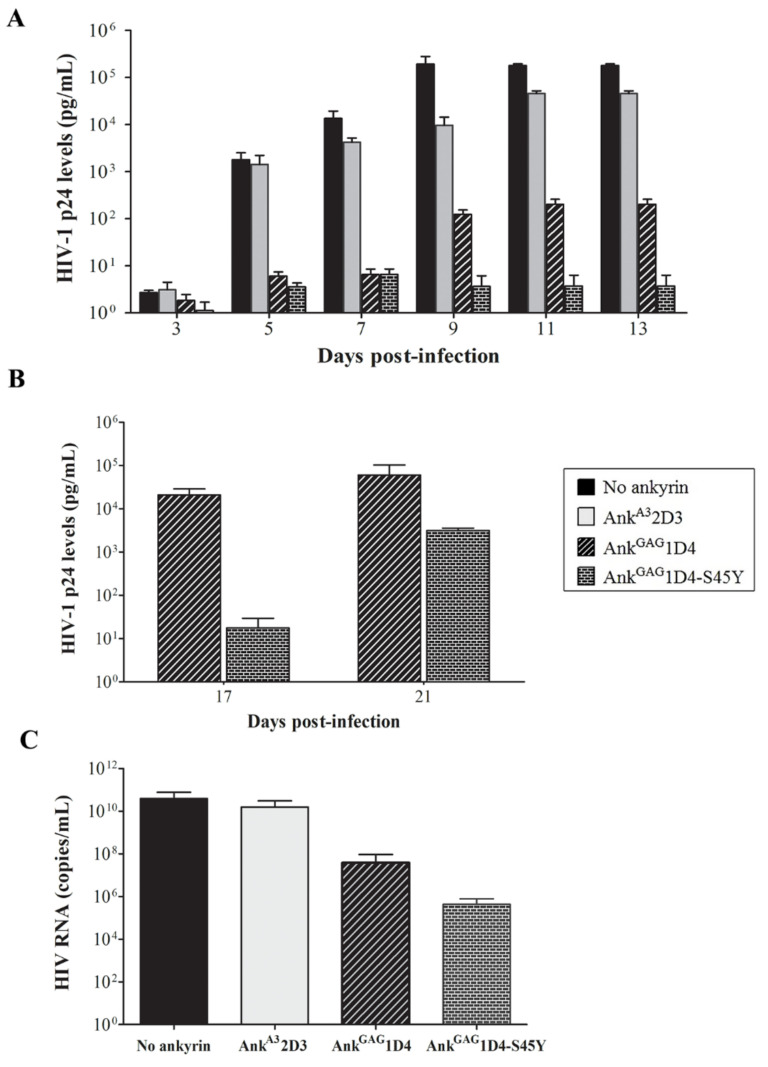
HIV replication in 50 MOI HIV-1-infected SupT1 cells expressing binding affinity-enhanced Ank^GAG^1D4. After HIV-1 challenge, cells were subcultured every 2 days. Cultured supernatant was collected at 3, 5, 7, 9, 11, 13, 17, and 21 days post-infection, then assayed to evaluate HIV-1 production. (**A**,**B**) HIV-1 p24 levels were determined using p24 ELISA. (**C**) HIV RNA copies were determined at 13 days post-infection using HIV viral load assay. Data represent mean ± SD from triplicate wells. No ankyrin, Ank^A3^2D3, Ank^GAG^1D4, and Ank^GAG^1D4-S45Y represent SupT1 cell control, SupT1 cells expressing Myr (+) Ank^A3^2D3-EGFP, Myr (+) Ank^GAG^1D4-EGFP, and Myr (+) Ank^GAG^1D4-S45Y-EGFP, respectively.

Furthermore, the anti-HIV-1 activity of Myr (+) Ank^GAG^1D4-S45Y-EGFP in more extensive infection was investigated. In this experiment, SupT1 cells and ankyrin-expressing SupT1 cells were infected with 50 MOI of WT HIV-1 NL4-3 virus and monitored as described above. Numerous syncytium cells were observed in SupT1 cell control and SupT1/Myr (+) Ank^A3^2D3-EGFP at 13 days post-infection (Figure S2). Myr (+) Ank^GAG^1D4-EGFP showed a delay in syncytium cell formation at 17 days post-infection, whereas Myr (+) Ank^GAG^1D4-S45Y-EGFP delayed the formation of syncytium cells to 21 days. HIV-1 p24 ELISA demonstrated a low level of HIV-1 p24 in SupT1 cells control at 3 days post-infection, then instantly increased at 5 days post-infection (Figure 6A). HIV-1 p24 was continuously detected at high levels in infected SupT1/Myr (+) Ank^A3^2D3-EGFP and produced similar levels to the SupT1 cell control. In contrast to Myr (+) Ank^GAG^1D4-EGFP and SupT1/Myr (+) Ank^GAG^1D4-S45Y-EGFP, HIV p24 was detected at low levels. At day 17 post-infection, the detected p24 level in SupT1/Myr (+) Ank^GAG^1D4-EGFP was 1.90 × 10^4^ pg/mL (Figure 6B), which slightly increased at day 21. Surprisingly, Myr (+) Ank^GAG^1D4-S45Y-EGFP retained the ability to inhibit HIV-1 replication. At 17 days post-infection, the p24 level in Myr (+) Ank^GAG^1D4-S45Y-EGFP was 1000 times lower compared with the parental ankyrin. The number of HIV RNA copies in SupT1/Myr (+) Ank^GAG^1D4-EGFP and SupT1/Myr (+) Ank^GAG^1D4-S45Y-EGFP was lower than in infected SupT1 cell controls and SupT1/Myr (+) Ank^A3^2D3-EGFP (Figure 6C). The number of HIV RNA copies was verified as 8.11 × 10^5^ copies/mL in SupT1/Myr (+) Ank^GAG^1D4-S45Y-EGFP and 1.02 × 10^7^ copies/mL in SupT1/Myr (+) Ank^GAG^1D4-EGFP. Viral load assay indicated that Myr (+) Ank^GAG^1D4-S45Y-EGFP performed better in blocking viral replication.

In addition, a single cycle assay was performed to investigate the role of Ank^GAG^1D4 and Ank^GAG^1D4-S45Y in inhibiting HIV-1 production. SupT1 cells and ankyrin-expressing SupT1 cells were infected with one MOI of VSV-G pseudotyped NL4-3 ∆Env virus (as shown in Supplementary Method). At 48 h post-infection, the morphology of infected SupT1 cells and ankyrin-expressing SupT1 cells was not different (Figure S3). In addition, HIV-1 p24 was determined by ELISA. The level of intracellular p24 of ankyrin-expressing SupT1 cells was not significantly distinct from controls. Whereas, extracellular p24 was decreased in ankyrin-expressing SupT1 cells (Figure S4A,B). The concentration of HIV-1 p24 in culture supernatant of SupT1/Myr (+) Ank^GAG^1D4-EGFP and SupT1/Myr (+) Ank^GAG^1D4-S45Y-EGFP was 28.78 and 8.94 pg/mL, respectively. These results suggested that HIV-1 assembly/release was impaired in the presence of ankyrin protein. Taken together, Ank^GAG^1D4-S45Y provides higher efficiency of intracellular antiviral effect on HIV-1 replication than the parental Ank^GAG^1D4.

### 3.4. Anti-HIV-1 Ankyrins Do Not Drive Mutation in Amino Acid Sequence of HIV-1 Capsid

**Figure 7:**
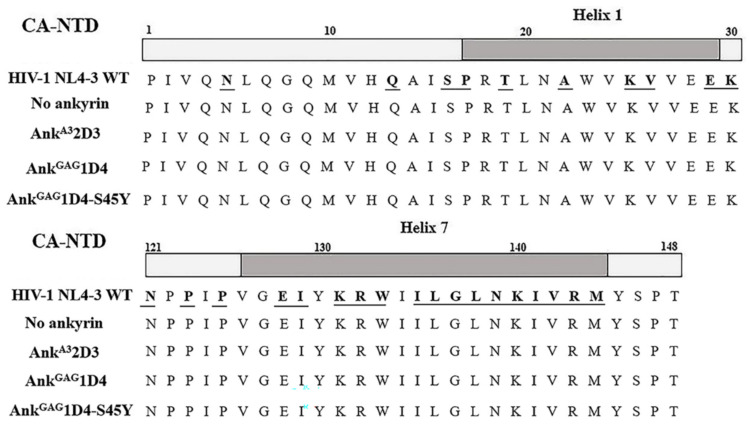
Sequencing analysis of the HIV-1 N-terminal capsid. WT HIV-1 NL4-3 viral RNA was extracted from culture supernatant harvested from HIV-1-infected cells. Then viral RNA was reverse transcribed into HIV-1 cDNA by RT-PCR. The HIV-1 capsid region was amplified and subjected to sequencing analysis. The diagram shows alignment of HIV-1 capsid sequence against WT HIV-1 NL4-3. Regions of helix 1 (upper) and helix 7 (lower) of HIV-1 capsid indicated in gray. Underlined letters indicate binding sites of ankyrins on the HIV-1 capsid. No ankyrin, Ank^A3^2D3, Ank^GAG^1D4, and Ank^GAG^1D4-S45Y represent HIV-1 capsid sequence of viral particles released from HIV-1 infected SupT1 cell controls, SupT1 cells expressing Myr (+) Ank^A3^2D3-EGFP, Myr (+) Ank^GAG^1D4-EGFP, and Myr (+) Ank^GAG^1D4-S45Y-EGFP, respectively.

According to the infection experiment, leakage of viral progeny was detected on the last day of observations. Therefore, we determined whether the leakage in protection was a result from mutation in the ankyrin-targeted region. Since our anti-HIV-1 ankyrins were against the HIV-1 capsid, viral cDNA was subjected to sequencing for capsid amino acid sequence analysis. According to the alignment result, no mutation was indicated, especially in helix 1 and helix 7 (Figure 7), targeting regions of ankyrin on the N-terminus capsid. These data suggest that the leakage of HIV-1 progeny was due to an overload of virus. In addition, Ank^GAG^1D4 and Ank^GAG^1D4-S45Y did not drive mutation in the HIV-1 capsid.

### 3.5. Binding Affinity-Enhanced Ankyrin Provides Antiviral Effects on HIV-1 Maturation Inhibitor Resistant Virus

**Figure 8:**
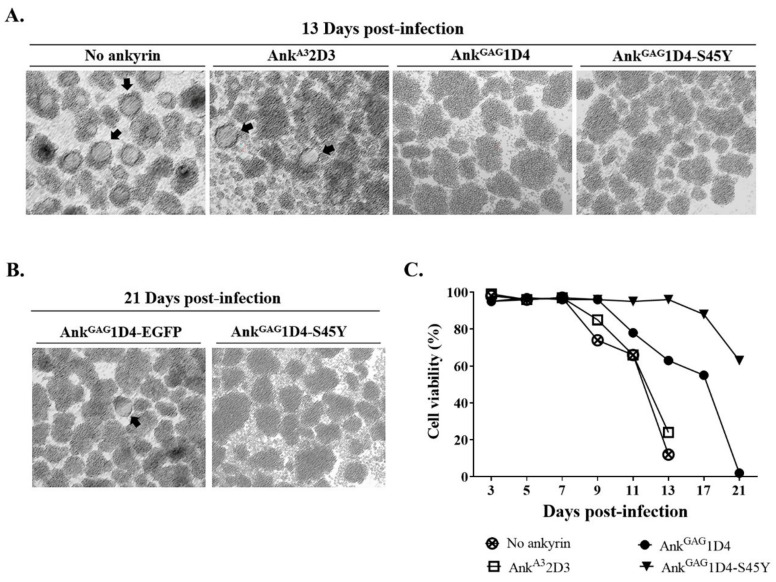
Cell morphology and cell viability of HIV-1 NL4-3 MIR_CAI201V_ infected SupT1 stable cells. SupT1cells and ankyrin-expressing SupT1 cells were infected with 10 MOI of HIV-1 MIR_CAI201V_ virus. After infection, cells were subcultured every 2 days. (**A**) Syncytium cells and cell morphology were observed under microscopy. Cell imaging was done at 10× magnification using Axio Vert.A1. (**B**) Cell morphology of infected SupT1/Myr (+) Ank^GAG^1D4-EGFP and SupT1/Myr (+) Ank^GAG^1D4-S45Y-EGFP was continuously observed until 21 days post-infection. Arrows point to syncytium cells. (**C**) Cell viability of infected cells was determined using Trypan blue exclusion method. No ankyrin, Ank^A3^2D3, Ank^GAG^1D4, and Ank^GAG^1D4-S45Y represent SupT1 cell control, SupT1 cells expressing Myr (+) Ank^A3^2D3-EGFP, Myr (+) Ank^GAG^1D4-EGFP, and Myr (+) Ank^GAG^1D4-S45Y-EGFP, respectively.

**Figure 9:**
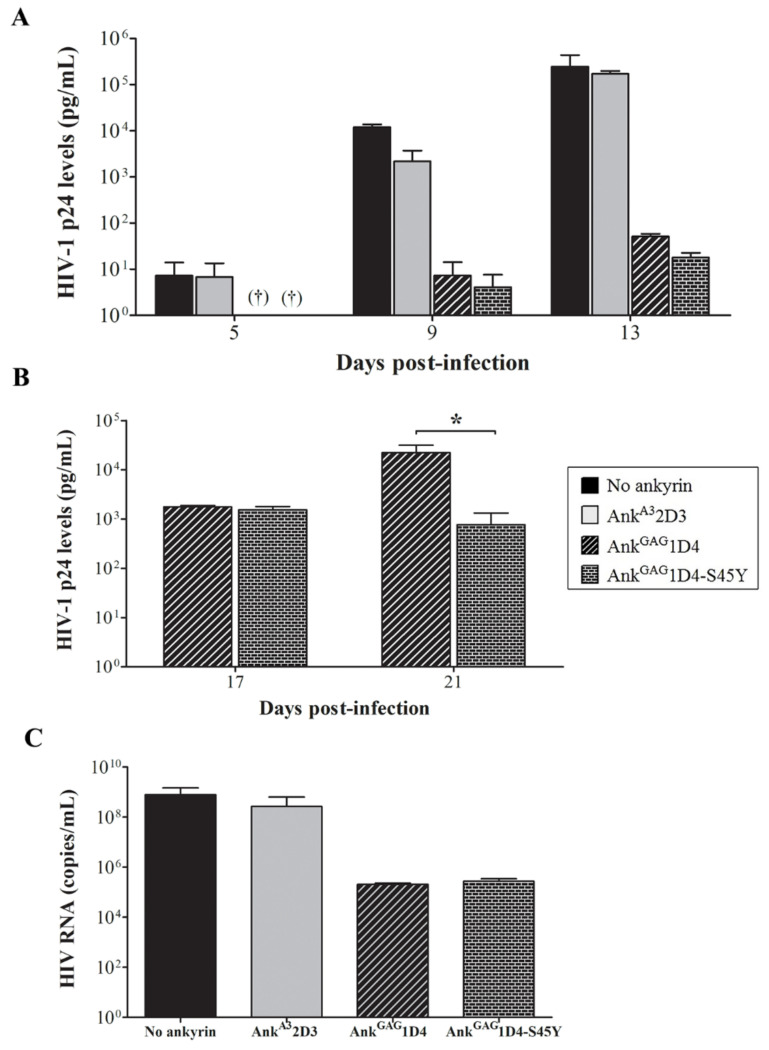
HIV-1 MIR viral replication in SupT1 cells expressing binding affinity-enhanced Ank^GAG^1D4. After HIV-1 challenge, cells were subcultured every 2 days. Cultured supernatant was collected at 5, 9, 13, 17, and 21 days post-infection, then assayed to evaluate HIV-1 production. (**A**,**B**) HIV-1 p24 levels were determined using p24 ELISA. † Indicates undetectable level of HIV-1 p24. (**C**) HIV RNA copies were determined at 13 days post-infection using HIV viral load assay. Data represent mean ± SD from triplicate well. ns, non-significant. * *p* ≤ 0.05 using unpaired *t*-test. No ankyrin, Ank^A3^2D3, Ank^GAG^1D4, and Ank^GAG^1D4-S45Y represent SupT1 cell control, SupT1 cells expressing Myr (+) Ank^A3^2D3-EGFP, Myr (+) Ank^GAG^1D4-EGFP, and Myr (+) Ank^GAG^1D4-S45Y-EGFP, respectively.

To solve the drug resistance issue, several anti-HIV-1 compounds were established; the HIV-1 maturation inhibitor is one anti-HIV-1 compound. Although these anti-HIV-1 compounds performed well in inhibiting HIV-1 production, a number of MI-resistant strains were reported. In this study, the antiviral activity of ankyrin on HIV-1 MIR virus was investigated. HIV-1 NL4-3 MIR_CAI201V_ was selected as a model to observe intracellular anti-HIV-1 activity of ankyrin. SupT1 cells and ankyrin-expressing SupT1 cells were infected with HIV-1 NL4-3 MIR_CAI201V_ virus at 10 MOI. After HIV-1 challenge, the infected cells were observed for syncytium formation under microscopy (Figure S5). Infected SupT1 cells and SupT1/Myr (+) Ank^A3^2D3 cells showed no protection against HIV-1 replication. A number of syncytial cells were observed on day 13 in SupT1 cells and SupT1/Myr (+) Ank^A3^2D3 cells with the appearance of clumping cells (Figure 8A). Consequently, p24 was detected at a very high level on day 13 (Figure 9A).

Both Myr (+) Ank^GAG^1D4 and Myr (+) Ank^GAG^1D4-S45Y expressed potency in inhibiting HIV-1 MIR virus. Interestingly, Myr (+) Ank^GAG^1D4-S45Y showed higher efficiency in protection, as syncytium cell formation was not observed until day 21 (Figure 8B), together with extended cell viability to day 21 (Figure 8C). Whereas, syncytium formation in Myr (+) Ank^GAG^1D4 was observed at day 17 post-infection (Figure S5). Consistently with the p24 level, the level of HIV-1 p24 in SupT1/Myr (+) Ank^GAG^1D4-S45Y was significantly lower than in infected SupT1/Myr (+) Ank^GAG^1D4 at 21 days post-infection (Figure 9B). However, HIV viral load assay indicated comparable anti-HIV-1 activity of Myr (+) Ank^GAG^1D4 and Myr (+) Ank^GAG^1D4-S45Y (Figure 9C). HIV-1 RNA copies in SupT1/Myr (+) Ank^GAG^1D4 and SupT1/Myr (+) Ank^GAG^1D4-S45Y were verified as 2.06 × 10^5^ and 2.61 × 10^5^ copies/mL, respectively.

## 4. Discussion

Although HAART is successfully used for HIV-1 therapy, it is limited by adverse drug effects and viral mutation. Furthermore, development of HIV-1 drugs takes years and is expensive [37]. It is desirable to establish new anti-HIV molecules against alternative viral targets in the HIV life cycle. Instead of antibodies and their derivatives, DARPins, representing a disulfide-independent scaffold, were sought for HIV-1 therapy based on their biological properties [3,38,39]. An extracellular DARPin, including CD4-specific DARPins and gp120-specific DARPins, was reported to inhibit HIV-1 entry [17,18]. Although these DARPins specifically perceive their target, limitations in terms of immune function and mutation-driven side effects were reported. CD4-specific DARPins can lead to impaired CD4 function, while gp120-specific DARPins drive mutation in the HIV-1 envelope. Moreover, a high clearance rate of DARPins in the blood circulation remains an obstacle [19]. Accordingly, we previously generated an intracellular Ank^GAG^1D4, which interferes with HIV-1 assembly by interacting with the N-terminus of HIV-1 capsid domain (CA-NTD) [21]. Several studies indicated that this specific area is crucial in viral assembly, maturation, and uncoating through viral capsid mutation [40,41]. The mutation leads to capsid polymorphisms that impair HIV-1 infectivity. Numerous CA-targeted molecules have been studied, such as PF74 [42], CAI [43], and GSCAI [16]. Although these molecules express activity in inhibiting HIV-1 replication, viral escape and inefficient cell penetration hamper its competency [44]. In contrast, DARPin is well-expressed inside the cells [45], specifically Ank^GAG^1D4, with the domain necessary for capsid polymorphism [21].

Despite the demonstrated anti-HIV-1 activity of Ank^GAG^1D4, protection in the late period of in vitro culture needs to be improved [24]. To enhance the efficiency of Ank^GAG^1D4, computational analysis was performed to identify the key residues on the ankyrin binding sites suitable for mutagenesis [25]. According to previous work, substituting tyrosine for serine improves the binding affinity of Myr (+) Ank^GAG^1D4-S45Y without altering specificity. However, an investigation of the intracellular anti-HIV activity of affinity-enhanced Ank^GAG^1D4 is required. In this study, antiviral activity of Myr (+) Ank^GAG^1D4-S45Y was observed in infected SupT1 cells compared with parental Myr (+) Ank^GAG^1D4. The affinity-improved ankyrin expression delayed the formation of syncytium cells and dramatically decreased viral replication as measured by p24 and viral load. By and large, different from mutant ankyrin, HIV-1 Gag was impaired in the assembly process, leading to reduced HIV-1 production in infected cells. The cytopathic effect in HIV-1 infected cells in the form of multinucleated giant cell formation and cell rupture represents a mechanism involving HIV-1 replication [46]. According to our experiment, inhibition of HIV-1 replication by Ank^GAG^1D4-S45Y results in late detection of syncytium formation, followed by extended cell viability. Although minimal progeny leakage was evidenced on day 21, the interactive amino acid sequence at HIV-1 NL4-3 CA was conserved. This suggests that both ankyrins were not likely to drive the mutation of HIV-1 NL4-3 CA. Thus, the progeny detected on the last day of culture probably resulted from the overload of HIV particles, which exceeded the ankyrin harness.

From our results, tyrosine substitution introduces binding affinity to intracellular Gag, since tyrosine is frequently found in the hot-spot of the antigen binding site [47]. Additionally, the role of tyrosine in mediating the binding of Ank^GAG^1D4 against a viral target has been highlighted by computational analysis and in vitro studies [25]. Tyrosine contains an aromatic side chain, comprising both a hydrophobic ring and a hydrophilic hydroxyl group, which contributes to its hydrogen bond forming ability, hydrophobic interaction, van der Waals interaction, and amino aromatic interaction [48,49]. Replacement with tyrosine provides more stable interaction of Ank^GAG^1D4-S45Y against viral targets, leading to higher efficiency in anti-viral activity.

Another group of CA-binding compounds is HIV-1 MIs. MIs confer anti-HIV activity through disrupting the maturation process of the virus at the CA–SP1 junction. However, HIV-1 is highly sensitive to mutations at the CA–SP1 junction, resulting in reports of MI-resistant strains [50]. Different from MI, our anti-assembly ankyrin specifically interacted with CA-NTD [21]. We assumed that Ank^GAG^1D4 inhibits replication of resistant viruses at the step prior to maturation. To prove the concept, we investigated the activity of Ank^GAG^1D4 and its mutant against MI-resistant virus. SupT1 cells expressing Ank^GAG^1D4 and Ank^GAG^1D4-S45Y were infected with HIV-1 NL4-3 MIR_CAI201V_ virus. This mutation has been reported to confer resistance against PF4696, the second-class HIV-1 maturation inhibitor, and partial resistance to BVM [33,34]. From the infection experiment, both anti-HIV-1 ankyrins were shown to have a negative effect on viral replication. Herein, HIV-1 NL4-3 MIR_CAI201V_ virus is a representative for observing antiviral activity against drug-resistant viruses. Another mutation on the HIV-1 capsid and downstream of the SP1 region also confers MI resistance [51,52]. Moreover, there are several reports on mutations in the HIV-1 genome, leading to the emergence of HIV-1 drug resistance against first-line ART [27] as well as a novel HIV-1 drug classes. For example, the M184V mutation along with thymidine analogue-associated mutations (TAMs) in HIV-1 reverse transcriptase gene increases abacavir resistance [53]. Another is Gag cleavage site mutation, which may confer resistance against protease inhibitors (PIs) in patients who fail PI-containing regimens [54]. As a different viral target site, Ank^GAG^1D4-S4Y might inhibit the viral replication of these resistant strains of HIV.

## 5. Conclusions

Our current results underscore the significance of Ank^GAG^1D4-S45Y for enhancing antiviral activity in either WT HIV-1 NL4-3 or MIR virus. Although the single amino acid change in a previous report did not markedly increase the affinity of Ank^GAG^1D4, the intracellular activity of Ank^GAG^1D4-S45Y demonstrated distinctly notable performance. Further improvement of Ank^GAG^1D4 affinity will provide a direction for rational design regarding predicted complexes from molecular dynamics (MD) simulations.

## Data Availability

Data are contained within the article or Supplementary Materials.

## Supplementary Materials

The following are available online at https://www.mdpi.com/article/10.3390/biom11101437/s1, Figure S1: Cell morphology of 10 MOI HIV-1 infected SupT1 cells at 5 days post-infection. Figure S2: Cell morphology of 50 MOI HIV-1 infected SupT1 cells. Figure S3: Cell morphology of 1 MOI of VSV-G pseudotyped NL4-3 ∆Env infected SupT1 cells or ankyrin-expressing SupT1 cells. Figure S4: Anti-HIV-1 activity of ankyrin proteins in VSV-G pseudotyped virus NL4-3 ∆Env infected SupT1 cells or ankyrin-expressing SupT1 cells. Figure S5: Cell morphology of 10 MOI HIV-1 MIR_CAI201V_ infected SupT1 cells.
