# Supplementary material for: Performance of Affinity-Improved DARPin Targeting HIV Capsid Domain in Interference of Viral Progeny Production
Source: Biomolecules. 2021 Sep 30;11(10):1437. doi: 10.3390/biom11101437 (PMC8533564; doi:10.3390/biom11101437)
Supplement: Supplementary file 1 [file biomolecules-11-01437-s001.zip › biomolecules-1381800-final-suppl/Supplementary files/Supplementary materials (final).pdf]

Supplementary Materials

Performance of Affinity-Improved DARPin Targeting HIV Capsid Domain in Interference of Viral Progeny Production

Kanokporn Sornsuwan[1,2], Weeraya Thongkhum[2,3], Thanathat Pamonsupornwichit[2], Tanawan Samleerat Carraway[4], Suthinee Soponpong[2,3], Supachai Sakkhachornphop[5], Chatchai Tayapiwatana[1,2,3*] and Umpa Yasamut[1,2,3*]

[1]Division of Clinical Immunology, Department of Medical Technology, Faculty of Associated Medical Sciences, Chiang Mai University, Chiang Mai 50200, Thailand; kanokporn_sornsuwan@cmu.ac.th (K.S.)
[2]Center of Biomolecular Therapy and Diagnostic, Faculty of Associated Medical Sciences, Chiang Mai University, Chiang Mai 50200, Thailand; thanathat.cmu@gmail.com (T.P.)
[3]Center of Innovative Immunodiagnostic Development, Faculty of Associated Medical Sciences, Chiang Mai University, Chiang Mai 50200, Thailand; weeraya.t@cmu.ac.th (W.T.), pear121000@gmail.com (S.S.)
[4]Division of Clinical Microbiology, Department of Medical Technology, Faculty of Associated Medical Sciences, Chiang Mai University, Chiang Mai 50200, Thailand; tsamleerat@gmail.com (T.S.C)
[5]Research Institute for Health Sciences, Chiang Mai University, Chiang Mai 50200, Thailand; ssakkhachornphop@yahoo.com (S.S.)
*Correspondence: chatchai.t@cmu.ac.th (C.T.), umpa.yas@cmu.ac.th (U.Y.)

Supplementary Method

Single cycle assay in VSV-G pesudotyped NL4-3 ΔEnv virus infected SupT1 cells and ankyrin expressing SupT1 cells

SupT1 cells and ankyrin-expressing SupT1 cells were infected with 1 MOI of VSV-G pseudotyped NL4-3 ΔEnv virus with an addition of 5 µg/ml of polybrene. Infected cells were spinoculated at 2,500 ×g, 32˚C for 1.30 h. After 16 hours post-infection, infected SupT1 cells and ankyrin-expressing SupT1 cells were washed three time with RPMI 1640 medium. Cells were resuspended with 10%-HI-FBS-RPMI 1640 medium, and cultured in humidified 5% CO_2 atmosphere incubator at 37 °C. At 48h post-infection, culture supernatants were collected, and centrifuged to remove debris and unwanted particles. Culture supernatant was kept at −80 °C for HIV-1 p24 ELISA. Additionally, cell pellets were harvested for determining intracellular HIV-1 p24.

Supplementary Figures

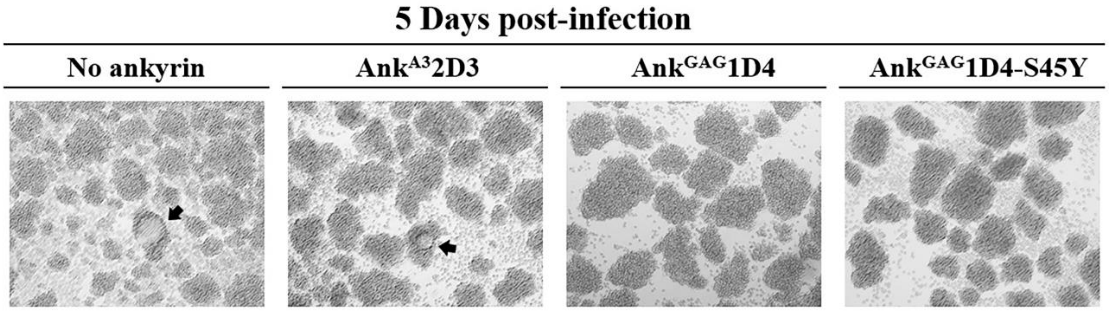

Figure S1. Cell morphology of 10 MOI of HIV-1-infected SupT1 cells expressing ankyrins at 5 days post-infection. Cell imaging was done at 20× magnification using Axio Vert.A1. Arrows point to syncytial cells. No ankyrin, Ank^{A3}2D3, Ank^{GAG}1D4, and Ank^{GAG}1D4-S45Y represent SupT1 cell control, SupT1 cells expressing Myr (+) Ank^{A3}2D3-EGFP, Myr (+) Ank^{GAG}1D4-EGFP, and Myr (+) Ank^{GAG}1D4-S45Y-EGFP, respectively.

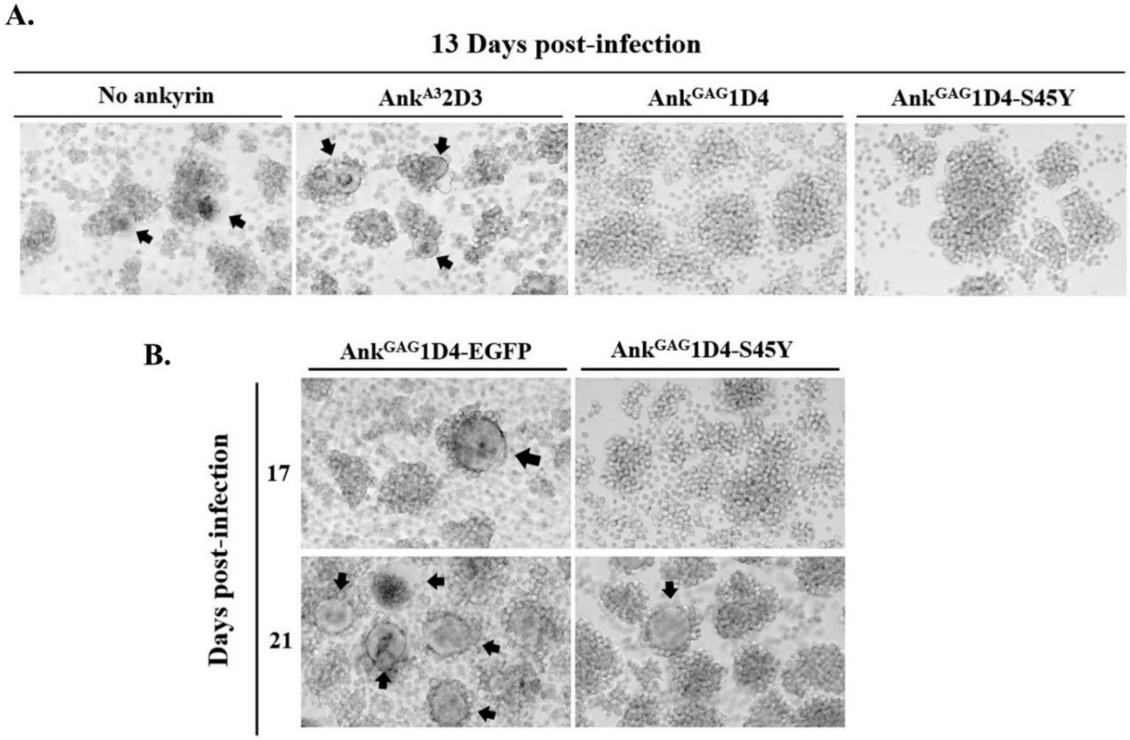

Figure S2. Cell morphology of 50 MOI HIV-1 infected ankyrin-expressing SupT1 cells. SupT1 cells and ankyrin-expressing SupT1 cells were infected with HIV-1 NL4-3 laboratory strain at 50 MOI. (A) Cell imaging was done at 20× magnification using Axio Vert.A1. Arrows point to syncytium cells. (B) Syncytium formation in infected SupT1/Myr (+) Ank^{GAG}1D4-EGFP and SupT1/Myr (+) Ank^{GAG}1D4-S45Y-EGFP was continuously observed until 21 days post-infection. No ankyrin, Ank^{A3}2D3, Ank^{GAG}1D4, and Ank^{GAG}1D4-S45Y represent SupT1 cell control, SupT1 cells expressing Myr (+) Ank^{A3}2D3-EGFP, Myr (+) Ank^{GAG}1D4-EGFP, and Myr (+) Ank^{GAG}1D4-S45Y-EGFP, respectively.

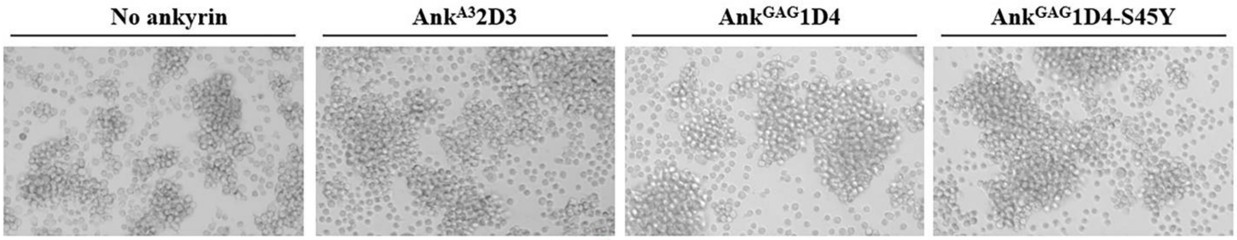

Figure S3. Cell morphology of 1 MOI of VSV-G pseudotyped NL4-3 ΔEnv infected SupT1 cells or ankyrin-expressing SupT1 cells. After 48 h post-infection, cell imaging was done at 20× magnification using Zeiss Colibri 7. No ankyrin, Ank^{A3}2D3, Ank^{GAG}1D4, and Ank^{GAG}1D4-S45Y represent SupT1 cell control, SupT1 cells expressing Myr (+) Ank^{A3}2D3-EGFP, Myr (+) Ank^{GAG}1D4-EGFP, and Myr (+) Ank^{GAG}1D4-S45Y-EGFP, respectively.

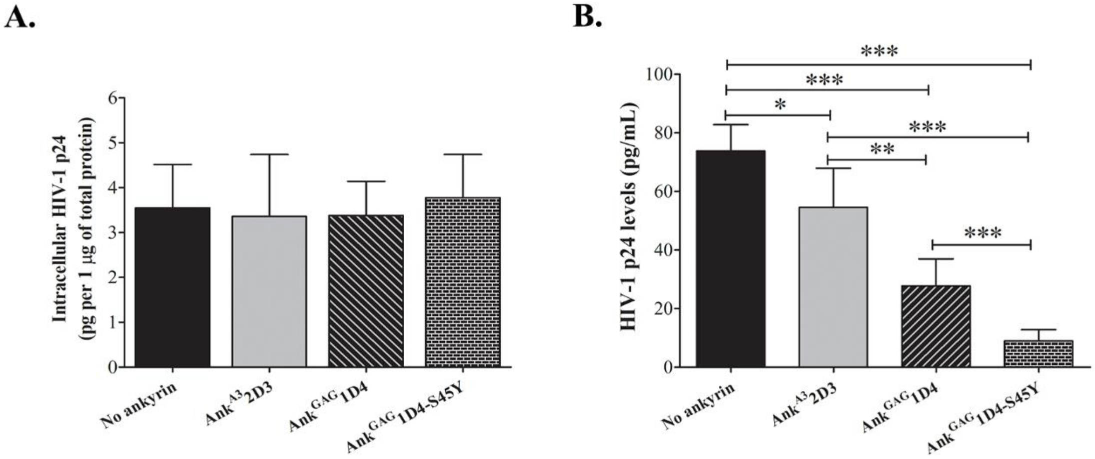

Figure S4. Anti-HIV-1 activity of ankyrin proteins in VSV-G pseudotyped NL4-3 ΔEnv infected SupT1 cells and ankyrin-expressing SupT1 cells. At 48h post-infection, cell pellet and culture supernatant were collected for evaluating HIV-1 p24 by ELISA. (A) Cells were lysed for detecting intracellular HIV-1 p24. The amount of HIV-1 p24 in 1 µg of total protein was demonstrated. (B) The level of HIV-1 p24 in culture supernatant was determined. Data represent mean ± SD from two independent experiment, triplicate wells each. * $p \leq 0.05$, ** $p \leq 0.01$ using unpaired t-test. No ankyrin, Ank^{A3}2D3, Ank^{GAG}1D4, and Ank^{GAG}1D4-S45Y represent SupT1 cell control, SupT1 cells expressing Myr (+) Ank^{A3}2D3-EGFP, Myr (+) Ank^{GAG}1D4-EGFP, and Myr (+) Ank^{GAG}1D4-S45Y-EGFP, respectively.

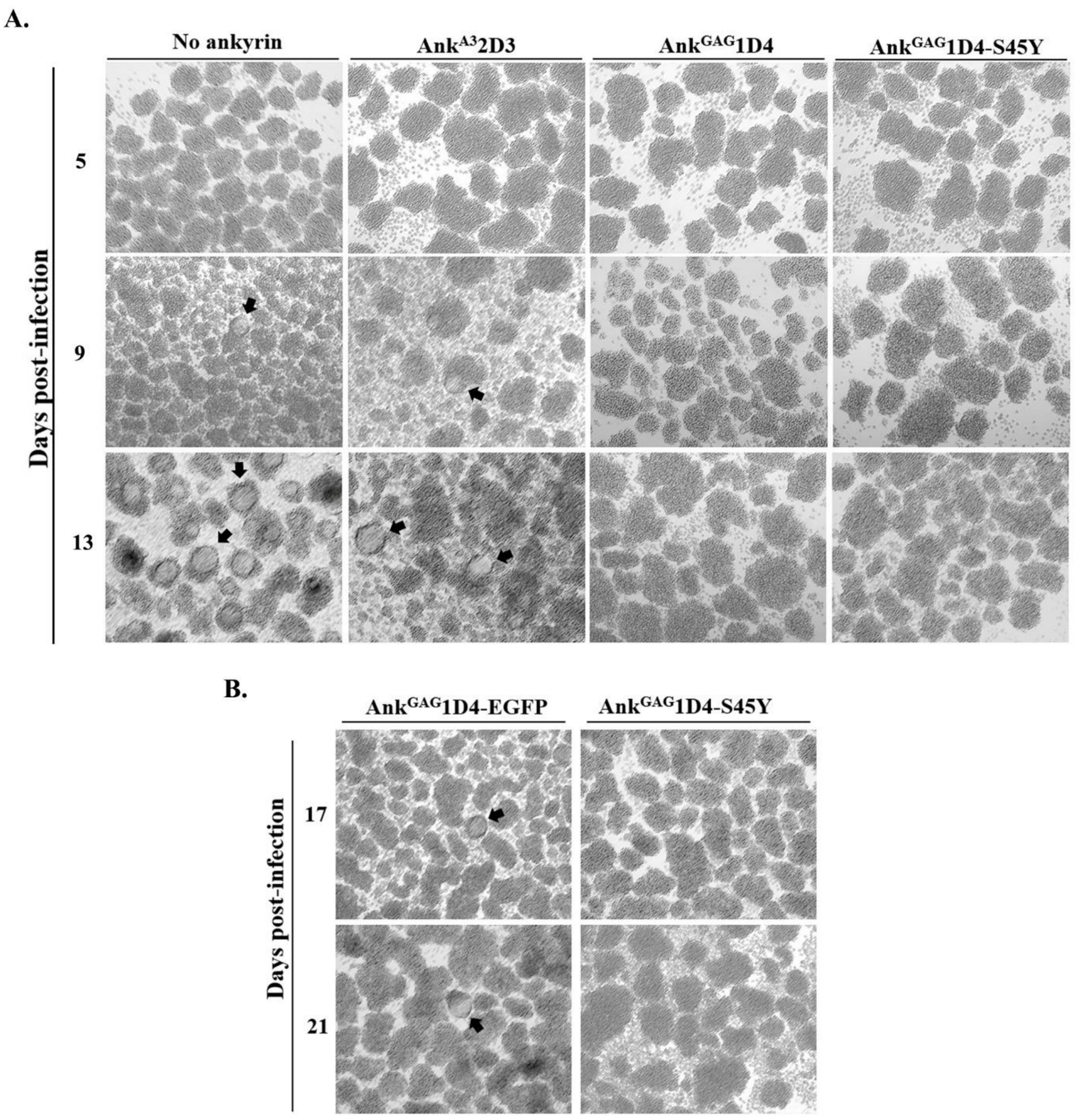

Figure S5. Cell morphology of HIV-1 NL4-3 MIR$_{CAI201V}$ infected SupT1 cells and ankyrin-expressing SupT1 cells were infected with 10 MOI of HIV-1 MIR$_{CAI201V}$ virus. After infection, cells were subcultured every 2 days, and syncytium cells and cell morphology were observed under microscopy. **(A)** Cell imaging was done at 10× magnification using Axio Vert.A1. **(B)** Morphology of infected SupT1/Myr (+) Ank^{GAG}1D4-EGFP and SupT1/Myr (+) Ank^{GAG}1D4-S45Y-EGFP was continuously observed until 21 days post-infection. Arrows point to syncytium cells. No ankyrin, Ank^{A3}2D3, Ank^{GAG}1D4, and Ank^{GAG}1D4-S45Y represent SupT1 cell control, SupT1 cells expressing Myr (+) Ank^{A3}2D3-EGFP, Myr (+) Ank^{GAG}1D4-EGFP, and Myr (+) Ank^{GAG}1D4-S45Y-EGFP, respectively.
