# Supplementary figures and images for: Performance of Affinity-Improved DARPin Targeting HIV Capsid Domain in Interference of Viral Progeny Production
Source: Biomolecules. 2021 Sep 30;11(10):1437. doi: 10.3390/biom11101437 (PMC8533564; doi:10.3390/biom11101437)

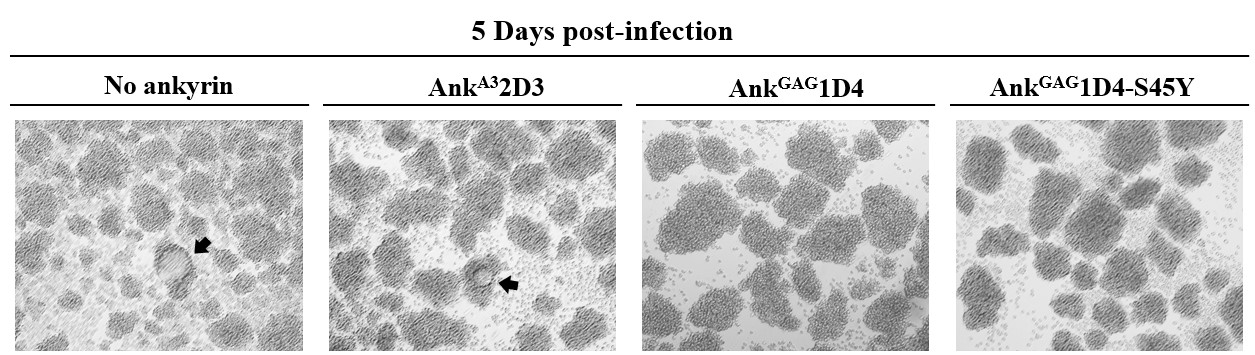

Supplement: Supplementary file 1 [file biomolecules-11-01437-s001.zip › biomolecules-1381800-final-suppl/Supplementary files/Figure S1/Figure S1.jpg]

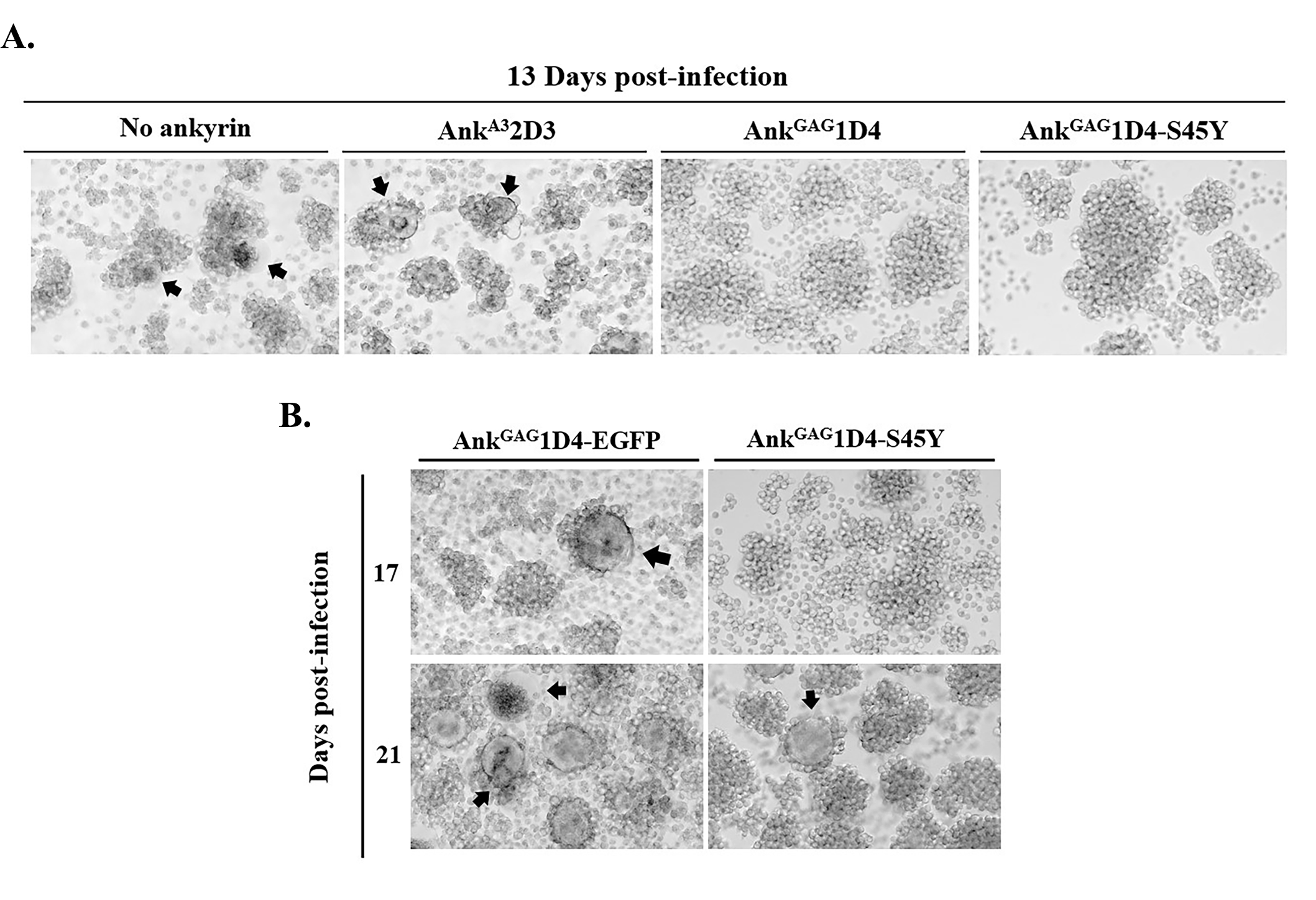

Supplement: Supplementary file 1 [file biomolecules-11-01437-s001.zip › biomolecules-1381800-final-suppl/Supplementary files/Figure S2/Figure S2..tif]

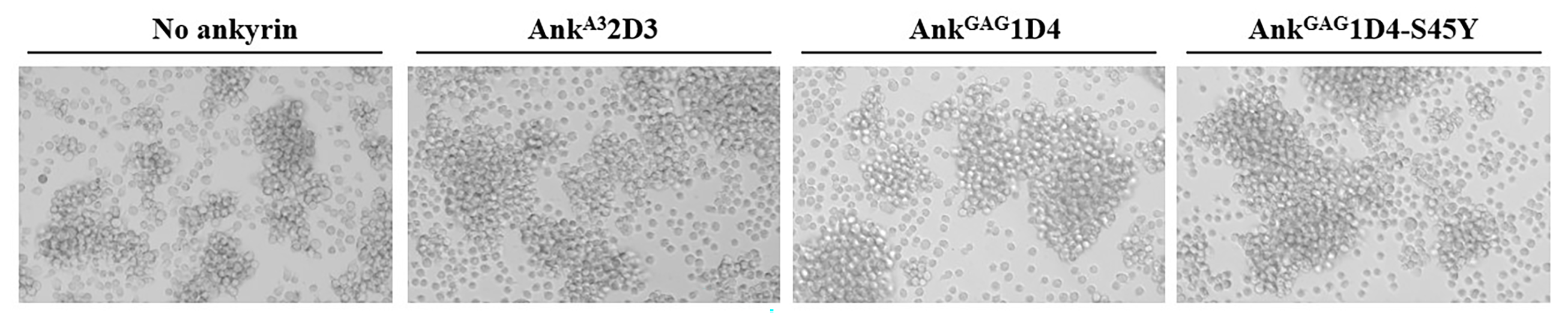

Supplement: Supplementary file 1 [file biomolecules-11-01437-s001.zip › biomolecules-1381800-final-suppl/Supplementary files/Figure S3/Figure S3.tif]

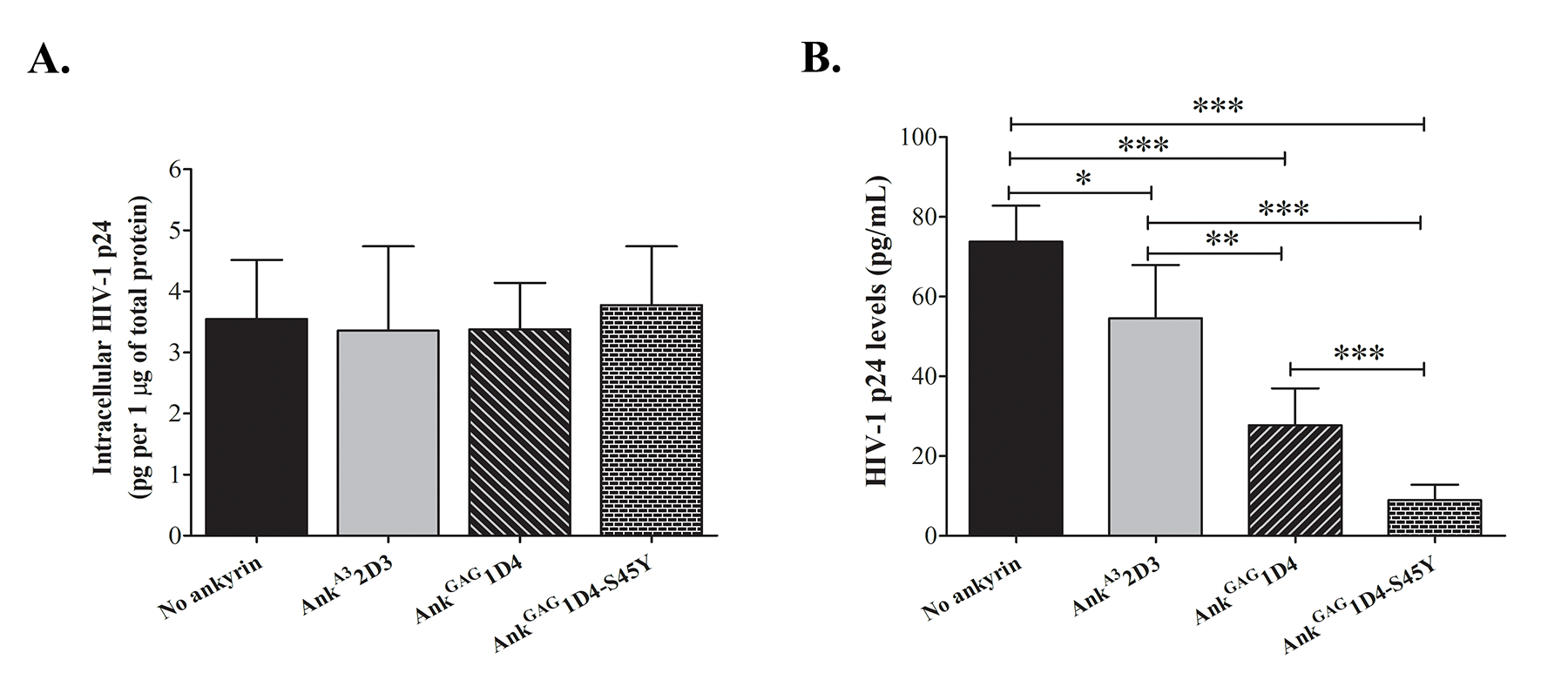

Supplement: Supplementary file 1 [file biomolecules-11-01437-s001.zip › biomolecules-1381800-final-suppl/Supplementary files/Figure S4/Figure S4.tif]

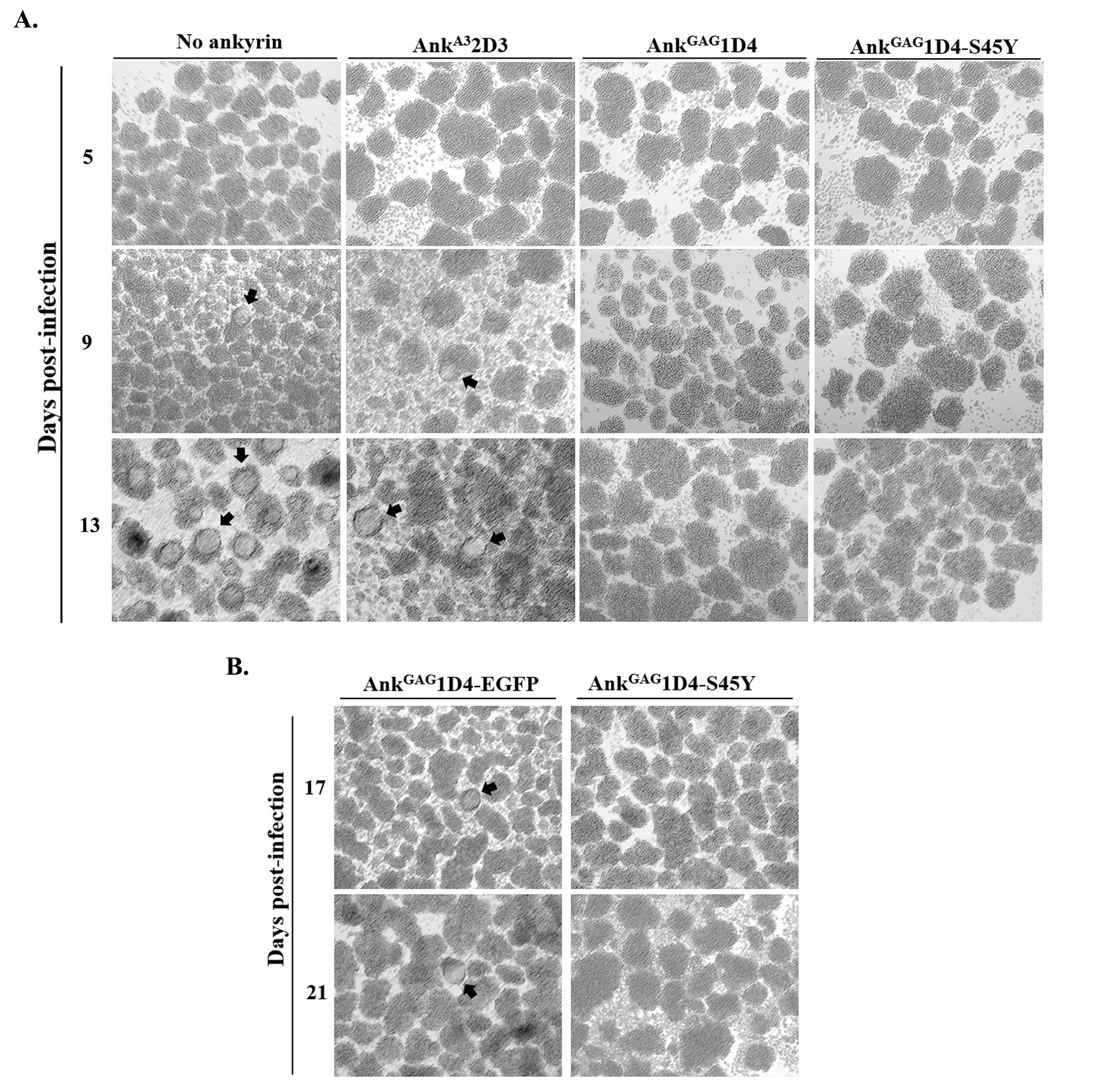

Supplement: Supplementary file 1 [file biomolecules-11-01437-s001.zip › biomolecules-1381800-final-suppl/Supplementary files/Figure S5/Figure S5.tif]
